# Influence of Sodium Chloride on the Behaviour of *Pseudomonas fluorescens* in Ripened Sheep Cheese

**DOI:** 10.3390/microorganisms13122693

**Published:** 2025-11-26

**Authors:** Simone Lopes, Manuela Vida, Cláudia Correia, Jaime Fernandes, Sandra Gomes, Ana Fernando, Rafael Tabla, Nuno Alvarenga

**Affiliations:** 1 NOVA School of Science & Technology, Universidade NOVA de Lisboa, 2829-516 Caparica, Portugal; jaime.fernandes@iniav.pt (J.F.); sandra.gomes@iniav.pt (S.G.); 2 Instituto Nacional de Investigação Agrária e Veterinária, 2780-157 Oeiras, Portugal; manuela.vida@iniav.pt (M.V.); claudia.correia@iniav.pt (C.C.); 3 GeoBioTec Research Center, NOVA School of Science & Technology, Universidade NOVA de Lisboa, 2829-516 Caparica, Portugal; 4 MEtRICs, QD, NOVA School of Science & Technology, Universidade NOVA de Lisboa, 2829-516 Caparica, Portugal; ala@fct.unl.pt; 5 LAQV-REQUIMTE, QD, NOVA School of Science & Technology, Universidade NOVA de Lisboa, 2829-516 Caparica, Portugal; 6 Centro de Investigaciones Científicas y Tecnológicas de Extremadura (CICYTEX), Área de Productos Lácteos, 06071 Badajoz, Spain; rafael.tabla@juntaex.es

**Keywords:** raw milk cheese, *Pseudomonas fluorescens*, sodium chloride and microbial spoilage

## Abstract

Ewe’s milk cheese produced from raw milk holds cultural and economic importance in Southern European countries; however, it poses microbiological challenges. Among spoilage microorganisms, *Pseudomonas fluorescens* is particularly concerning due to thermostable enzymes that impair the texture, aroma, and stability of cheese, even under refrigeration and salinity. This study evaluated the influence of sodium chloride concentration on *Pseudomonas fluorescens* given the pivotal role of salt in ensuring cheese stability and safety. Cheeses inoculated with *Pseudomonas fluorescens* were produced under an experimental design that combined three ripening temperatures with four salt concentrations. Physicochemical composition and microbiological stability were assessed at the end of ripening (20 days). Results showed that the ripening temperature emerged as the most determinant factor, influencing microbial viability and increasing solid retention, proteolysis, and dehydration, leading to harder cheeses. Low temperatures without salt favoured surface colour defects, whereas, although high salt levels contributed to partial control of *Pseudomonas* spp., they also delayed ripening, resulting in cheeses with a pale, uncharacteristic appearance. Conversely, moderate salinity (2%) combined with higher ripening temperatures promoted uniform maturation, resulting in a stable texture and appearance free of defects. These findings highlight the need to balance salt and ripening conditions to optimise quality and safety in traditional raw ewe’s milk cheeses.

## 1. Introduction

The production of ewe’s milk cheese from raw milk represents one of the most authentic and historically preserved expressions of Mediterranean cheesemaking traditions. In Portugal, raw ewe’s milk cheeses such as Serra da Estrela, Serpa, Castelo Branco, Évora, and Azeitão are emblematic products protected under the European PDO status, which ensures their traceability, sensory typicity, and cultural value [1,2,3]. These cheeses are not only gastronomic icons but also play a crucial economic role for artisanal producers and rural communities, linking local biodiversity and traditional know-how to territorial valorisation [4]. Their distinctive sensory profiles result from the synergy between the biochemical composition of raw ewe’s milk—characterised by high protein, fat, and mineral content—and the activity of native microbiota, endogenous enzymes, and specific ripening conditions [5,6,7].

The authenticity of raw ewe’s milk cheeses is strongly linked to the presence of a diverse native microbiota that actively drives biochemical transformations during ripening. Lactic acid bacteria (LAB) play a central role by ensuring rapid acidification, inhibiting pathogens and spoilage organisms, and initiating proteolysis and lipolysis, which release peptides, amino acids, and free fatty acids essential for flavour development [1,3,8,9]. Yeasts and moulds, such as *Debaryomyces hansenii* and *Geotrichum candidum*, also contribute to lipolysis, surface pH modulation, and the production of volatile aromatic compounds [10,11]. This microbial ecosystem is shaped by factors such as milk composition, hygiene practices, environmental sources (e.g., wooden boards and brines), and ripening conditions, which collectively promote a succession of microbial communities over time [6,12]. Such dynamics confer cheeses with their unique sensory identity but also create vulnerabilities, since the same microbial complexity that enhances authenticity may enable the survival of undesirable species. Several studies have reported high microbial richness in raw milk cheeses, including LAB, Gram-positive catalase-positive bacteria, Gram-negative psychrotrophs, yeasts, and moulds, all of which interact in a competitive ecological network that can either stabilise or destabilise the product [3,13]. Beyond their technological functions, some LAB and yeast strains exhibit probiotic and biofunctional potential, with reported antioxidant, immunomodulatory, and anti-inflammatory properties [14,15]. However, this beneficial potential coexists with microbiological risks: the absence of pasteurisation allows persistence of spoilage bacteria and occasionally pathogens, requiring careful management of ripening conditions to balance sensory quality and safety [13,16].

The psychrotrophic *Pseudomonas* spp. are particularly concerning in the dairy sector due to their ability to proliferate under refrigeration and produce thermostable proteases and lipases that remain active after pasteurisation [17,18,19]. The AprX protease, for instance, retains significant activity even at 7 °C, leading to casein degradation and the release of hydrophobic peptides associated with bitterness [20,21]. In parallel, lipases hydrolyse triglycerides into short-chain fatty acids, generating rancid or soapy flavours [22]. These alterations not only impair sensory quality but also reduce yield and interfere with acidification by competing with LAB for substrates [23]. *Pseudomonas fluorescens*, in particular, is widely reported in raw milk, storage tanks, and ripened cheeses, where it persists through biofilm formation on industrial surfaces, increasing resistance to cleaning and disinfection [24,25]. In addition, its ability to secrete siderophores such as pyoverdine enhances competitiveness against LAB, while pigment production (e.g., pyocyanin, pyomelanin, and yellow or blue chromophores) causes visible defects that often lead to product rejection [26]. Together, these traits make *Pseudomonas fluorescens* one of the most relevant spoilage bacteria in raw milk cheeses and a critical target for preventive strategies.

Recent next-generation sequencing studies have provided a deeper understanding of the microbial ecology of raw ewe’s milk cheeses. These investigations consistently report *Lactococcus lactis*, *Leuconostoc mesenteroides*, *Lactobacillus plantarum*, and *Enterococcus faecalis* as dominant LAB driving acidification and flavour development, together with yeasts such as *Debaryomyces hansenii* and *Geotrichum candidum* and filamentous fungi belonging mainly to *Penincillium* spp. The relative abundance of these microorganisms is strongly affected by ripening temperature, salt concentration, and environmental microbiota, which collectively shape the maturation process [1,11,27,28].

Sodium chloride (NaCl) is one of the oldest and most influential technological agents in cheesemaking, exerting multiple roles on microbial ecology, enzymatic activity, texture, and flavour [29,30]. By reducing water activity, NaCl creates an unfavourable environment for Gram-negative bacteria and moulds, historically justifying its widespread preservative use [31]. At the same time, salt modulates biochemical reactions, slowing lactose fermentation and pH decline, while influencing proteolysis, lipolysis, and the release of volatile aroma compounds [6,32]. Moderate concentrations of NaCl (≈2–4%) are generally considered optimal to balance microbiological stability and sensory complexity [33]. However, consumer-driven initiatives to reduce sodium intake, given that global salt consumption averages 9 g/day, well above the WHO recommendation of 5 g/day, pose new challenges for the safety and acceptance of traditional cheeses [34]. Lower salt levels increase water activity, accelerate proteolysis, and may release bitter peptides, jeopardising sensory quality [35]. Moreover, *Pseudomonas fluorescens* can tolerate 2.5–3.5% NaCl through adaptive mechanisms such as biofilm formation and ion transporters, questioning the adequacy of salt alone as a barrier [36].

Overall, the literature underscores the dual nature of raw ewe’s milk cheeses; while their authenticity relies on the activity of native microbiota, they remain vulnerable to spoilage by *Pseudomonas fluorescens*, and the role of salt in controlling these dynamics is complex and not fully understood. Although the technological importance of NaCl in cheese has been extensively studied, the combined influence of sheep’s milk cheeses had not been systematically addressed. Against this background, the present study was designed to evaluate the combined effects of NaCl concentration and ripening temperature on the behaviour of *Pseudomonas fluorescens* and on the microbiological, physicochemical, and structural evolution of raw ewe’s milk cheese. By integrating microbial counts, biochemical traits, texture, and colour analysis through response surface methodology and multivariate statistics, this work aims to provide insights into strategies that reconcile sodium reduction with the preservation of authenticity, safety, and quality in artisanal cheeses.

## 2. Materials and Methods

### 2.1. Experimental Design

A factorial design was applied to evaluate the effect of salt concentration and ripening temperature on the survival of *Pseudomonas fluorescens* in traditional ewe’s milk cheese. Four salt concentrations (0%, 2%, 4%, and 6%) and three ripening temperatures (8 °C, 11 °C, and 14 °C) were tested. Raw ewe’s milk was inoculated with a defined concentration of *Pseudomonas fluorescens* prior to cheesemaking to ensure consistent contamination across treatments. For each experimental condition, three independent cheeses were produced as biological replicates. Raw ewe’s milk used for cheesemaking was analysed for its initial microbiological and physicochemical characteristics. Cheese samples were collected after 20 days of ripening, which represented the main time point for analysis in this study.

### 2.2. Bacterial Strains and Culture Conditions

The *Pseudomonas fluorescens* strains used in this study were isolated within the TID4AGRO (INTERREG) project from Portuguese artisanal raw ewe’s milk cheeses. Colonies showing typical *Pseudomonas* spp. traits (pigmentation, fluorescence under UV light, oxidase positive, and glucose non-fermentative) were selected and preserved at −80 °C in a mix of 50% glycerol and H_2_O and 50% Tryptic Soy Broth (TSB).

For reactivation, isolates were grown in TSB at 30 °C during 24 h and streaked onto Tryptic Soy Agar (TSA). Molecular identification by partial sequencing of gyrB, rpoD, and rpoB genes confirmed the species as *Pseudomonas fluorescens* (>99% identity) [37].

Bacterial suspensions were prepared from TSA colonies resuspended in sterile saline (0.85% NaCl) and adjusted to McFarland 5 (1.5 × 10^9^ CFU/mL), verified with a Densimat (bioMérieux, Lyon, France). The suspension was freshly prepared before inoculation.

### 2.3. Cheese Production

Raw ewe’s milk was supplied by Ferrolho & Ferrolho, Lda. (Beja, Portugal), transported under refrigeration (4 °C) in washed and sanitised containers, and processed within 24 h of arrival. From the total volume received, aliquots were reserved for initial microbiological and physicochemical analyses.

Cheesemaking was carried out in thermostatic vats (Armfield Cheese Vat FT20, Hampshire, UK) containing 9 L of raw milk each, inoculated with 0.6 L of the prepared *Pseudomonas fluorescens* suspension. Milk was kept at 30 °C and allowed to incubate for 1 h, followed by addition of rennet, 2 mL Maxiren^®^ 180 liquid coagulant (Dsm-firmenich, Maastricht, The Netherlands) per vat, and 30 min coagulation. The curd was cut in two steps (vertical and horizontal) with intermediate resting time (10 min) and stirred for whey expulsion. Salt was incorporated directly into the curd according to the experimental concentrations: 0%, 2%, 4%, and 6%.

After moulding and a 45 min resting period at room temperature, cheeses were ripened for 20 days at 8 °C, 11 °C, and 14 °C, with the relative humidity maintained between 85 and 90%. Cheeses were turned daily and, on the 10th day, all cheeses were washed with saline solution matching their treatment to control surface flora and promote uniform rind development.

### 2.4. Physicochemical Analyses

Raw milk was initially characterised using a MilkoScan™ Mars (Foss Electric, Hillerød, Denmark), according to ISO 21543:2020 [38]. In addition, pH was measured using a pH meter (Metrohm 713, Herisau, Switzerland); titratable acidity and NaCl content were determined in milk according to NP 470:1983 [39] and NP 471:1985 [40]. For cheeses, the following determinations were performed: pH was evaluated with a penetration electrode at 20 ± 1 °C [41], titratable acidity according to AOAC 920.124 [42], NaCl content according to ISO 5943 [43], fat content according to ISO 3433:2008 [44], and dry matter according to NP 3544:1988 [45].

Nitrogen content was determined by the Kjeldahl method using a digestion block (Tecator, 2020 Digestor, Foss, Hillerød, Denmark) and steam distillation with a colorimetric endpoint detection (Kjeltec 2300 Analyzer Unit, Foss, Hillerød, Denmark); water soluble nitrogen (WSN) and trichloroacetic acid-soluble nitrogen (TCA-SN) were determined according to ISO 8968-1&2|IDF 20-1&2 and AOAC 920.123 [46,47]. Protein was quantified applying a conversion factor of 6.38; this factor is the standard one recommended for milk and dairy products and is based on the average nitrogen content of milk proteins [48]. WSN was obtained after aqueous extraction of nitrogenous compounds, followed by the Kjeldahl method. TCA-SN was quantified through N-component precipitation using a 12% trichloroacetic acid solution, followed by nitrogen determination in the filtrate (Whatman No. 42 filter paper) employing the Kjeldahl method [38]. Texture was evaluated using a texture analyser (TAHDi, Stable Micro Systems Ltd., Godalming, UK), and colour was assessed in the CIELab system (L*, a* and b*), using a colorimeter (Chorma Meter CR:330, Minolta, Osaka, Japan).

### 2.5. Microbiological Analyses

Ten grams of each cheese was aseptically transferred into sterile bags containing 90 mL of sterile 2% (*w*/*v*) sodium citrate solution and homogenised in a Stomacher 400^®^ (Lab Blender, model BA 7021, Seward Medical, London, UK) for 1 min to obtain the $10^{-1}$ dilution. Serial decimal dilutions were prepared in Ringer’s solution, and appropriate dilutions were prepared according to each salt concentration previously defined. All analyses were performed under aseptic conditions, and colony counts were expressed as log colony-forming units per gram of sample (CFU/g).

LAB were enumerated according to ISO 15214:1988 [49], using de Man, Rogosa and Sharpe (MRS) agar by pour plate method, incubated at 30 °C for 72 h. Yeasts and moulds were quantified according to ISO 21527-1:2008 [50], on Dichloran Rose Bengal Chloramphenicol Agar (DRBC) incubated at 25 °C for 120 h. *Pseudomonas* spp. were enumerated following ISO 13720:2010 [51], using Cetrimide–Fucidin–Cephaloridine (CFC) agar supplemented with CFC selective supplement (Oxoid SR0103E), incubated at 25 °C for 48 h.

### 2.6. Statistical Analysis

Descriptive statistics (means, standard deviations, and 95% confidence intervals) were calculated for all physicochemical and microbiological parameters. Each condition was tested in triplicate (*n* = 3).

All data were analysed using a one-way Analysis of Variance (ANOVA) with significance set at *p*-value (*p*) < 0.05. Mean comparisons were performed with the Scheffé post hoc test. All results were presented in tables in the following format: mean (standard deviation) ^significance^. Response surface methodology was applied to model the combined effect of salt concentration and ripening temperature on *Pseudomonas fluorescens* counts. Principal component analysis (PCA) was performed on a set of physicochemical and microbiological parameters to explore grouping patterns among cheeses after 20 days of ripening [41].

All statistical analyses were conducted using Statistica v12.5 for Windows (StatSoft, Inc., Tulsa, OK, USA).

## 3. Results

Results are presented sequentially, beginning with the characterisation of raw ewe’s milk, followed by microbial dynamics, physicochemical composition, protein fractions, texture, and colour, under the combined influence of salt concentration and ripening temperature. This framework provides an integrated overview of the technological and microbiological changes occurring over 20 days of ripening.

Table 1 summarises the physicochemical and microbiological parameters of the raw ewe’s milk used for cheesemaking, providing the baseline composition and microbial load, prior to inoculation and ripening.

As shown in Table 1, the mean pH was 6.62 and the titratable acidity 18.0 mL NaOH 0.1 N/L, consistent with reported ranges for ovine milk [52]. Fat (6.21%) and lactose (4.95%) were within expected values [5], whereas protein (2.52%) and total solids (13.80%) were lower than average literature values (≈5–6% protein and ≈18% total solids), likely reflecting seasonal or animal-related variation [5,52]. The cryoscopic point (−0.516 °C) also indicated reduced non-fat solids [53]. Microbiologically, LAB counts were moderate (4.38 log CFU/g), while *Pseudomonas* spp. reached unusually high levels (7.13 log CFU/g), exceeding typical values for raw ovine milk (≈3 log CFU/g) [54] and approaching those reported after refrigerated storage (3–7 log CFU/g) [55].

**Figure 1:**
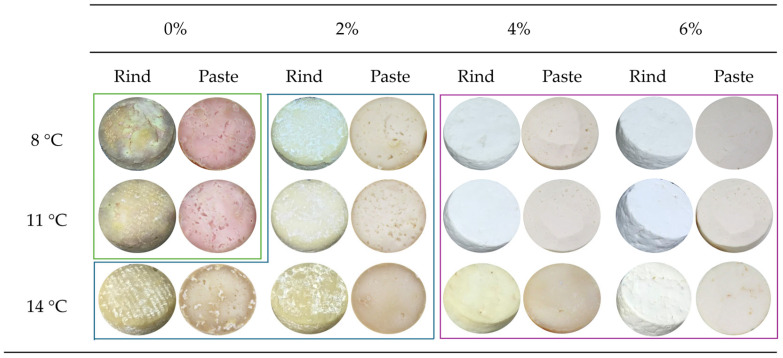
Cheese samples after 20 days of ripening at 8 °C, 11 °C, and 14 °C, with different salt concentrations: 0%, 2%, 4%, and 6%.

Figure 1 presents the visual impact of varying salt concentrations and ripening temperatures on cheese appearance after 20 days of maturation. Distinct visual patterns were observed, enabling the classification of samples into three groups with characteristic surface and structural features.

The visual analysis of the samples allowed their grouping into three distinct categories: (i) the first group (green lines) comprised the samples without added salt, ripened at 8 °C and 11 °C, which exhibited pronounced chromatic defects attributed to the activity of *Pseudomonas* spp. (ii) The second group (blue lines) included the samples produced with 2% salt, as well as the salt-free sample ripened at 14 °C, which displayed typical characteristics of this type of cheese, namely a smooth and well-formed rind, straw-yellow colouration, regular shape with slight lateral bulging, and, in some cases, the presence of superficial white moulds [56]. (iii) The last group (pink lines) consisted of the sample with 4% and 6% of salt, which were distinguished by a whitish colouration and the formation of sharp edges, features that are not characteristic of ripened cheeses of this typology.

Table 2 presents the mean log counts of *Pseudomonas* spp., LAB, and yeasts/moulds in cheeses after 20 days of ripening under different combinations of salt concentration and temperature. After 20 days of ripening, *Pseudomonas* spp. remained at elevated levels in most treatments (≈8.5–9.3 log CFU/g), confirming their resilience under saline and refrigerated conditions. The lowest counts were observed at 14 °C with 2% salt (6.58 log CFU/g), suggesting a stronger inhibitory effect when moderate salinity was combined with higher ripening temperature [16,33]. LAB reached consistently high populations (>8.0 log CFU/g), with maximum levels recorded at 8 °C and 4% salt (8.98 log CFU/g). This highlights their ecological advantage during ripening, even under elevated salt concentrations, supporting their role as a natural barrier against spoilage microorganisms [8,54].

Yeasts and moulds also developed substantially, ranging from 5.55 log CFU/g (11 °C, 0% salt) to 9.05 log CFU/g (8 °C, 2% salt). Their proliferation confirms the relevance of yeasts and moulds in the later stages of ripening, where they contribute to lipolysis and flavour development but may also pose risks when present in excessive amounts [28].

After 20 days of ripening, Table 3 shows that cheese pH ranged from 4.91 (11 °C, 4% salt) to 6.25 (14 °C, 0% salt). Higher salinity generally resulted in lower pH, consistent with salt’s inhibitory effect on LAB metabolism, whereas the absence of salt led to incomplete acidification [8,29]. Titratable acidity ranged from 7.20 mL NaOH 0.1N/100 g (8 °C, 2% salt) to 16.27 mL NaOH 0.1N/100 g (11 °C, 0% salt). The highest acidity was associated with cheeses with no salt added, while increasing NaCl reduced acid development. This trend aligns with previous studies highlighting the moderating effect of salt on lactose fermentation [26,29,32].

Fat content was strongly influenced by ripening temperature, with cheeses matured at 14 °C retaining higher fat (32–39%), while those at 8 °C and 11 °C remained below 30%. This pattern reflects reduced syneresis and greater solid retention at higher temperatures [35]. Total solid content followed a similar trend, with maximum values at 14 °C (≈68–72%) and the lowest levels at 8 °C and 11 °C, particularly at 6% salt (≈47–49%). This indicates a strong effect of temperature on water loss and solid concentration, modulated by salt [14,16].

Table 4 shows the levels of total nitrogen (TN), protein content, and nitrogen fractions in ripened cheeses, allowing assessment of proteolysis under the different experimental conditions. After 20 days of ripening, TN values ranged from 2.70% (11 °C, 6% salt) to 5.13 (14 °C, 0% salt), reflecting the combined influence of salinity and ripening temperature on nitrogen retention [29,52]. The WSN/TN % varied between 20.22% (14 °C, 6% salt) and 55.99% (8 °C, 0% salt). Likewise, the proportion of TCA-SN/TN % ranged from 2.24% (14 °C, 6% salt) to 21.85% (8 °C, 0% salt). Both indicators demonstrated that proteolysis was more intense in unsalted cheeses at lower to intermediate ripening temperatures (8 °C and 11 °C), while higher temperatures (14 °C) promoted greater protein retention but comparatively lower relative proteolysis.

The hardness values, as shown in Table 5, were generally low (<5 N) at 8 °C and 11 °C, confirming a softer, less compact texture. In contrast, cheeses ripened at 14 °C showed a sharp increase, peaking at 48.7 N (14 °C, 2% salt). The effect of salt was not linear, since excessive levels (6%) led to lower firmness, in line with previous reports on casein network disruption at high salinity [57,58].

Force values followed a similar trend, with modest resistance at 8 °C and 11 °C (≈ 28–47 N) and markedly higher at 14 °C, reaching up to 401.5 N (14 °C, 6% salt). This reflects greater solid retention and matrix compaction at higher temperatures [59,60].

Adhesiveness ranged from 9.1 − Ns (8 °C, 4% salt) to 158.6 − Ns (14 °C, 6% salt). Strongly adhesive textures occurred only at 14 °C, suggesting enhanced water and fat retention under these conditions [57].

For the rind, L* values fluctuated slightly without a clear pattern, linked to salt or temperature, suggesting the influence of intrinsic milk factors or surface reactions [59]. In contrast, a* values were more positive at 8 °C and 11 °C and shifted to more negative values at 14 °C, indicating that higher temperature may reduce red intensity through pigment oxidation [33]. As for b* values, the highest readings were recorded at 14 °C, confirming stronger yellow tones at elevated ripening temperature [61].

In the cheese paste, L* was lower at 11 °C and 14 °C, particularly with added salt, but no consistent trend emerged. The a* coordinate was always negative, with the strongest greenish tones at 14 °C, suggesting temperature had greater impact than salinity; temperatures (11 °C) showed the lowest intensity.

### 3.1. Response Surface Analysis (RSM)

To further study the interaction between ripening temperature and salt concentration, RSM was applied. Multiple regression models were fitted for microbiology and physicochemical parameters. A three-dimensional response surface was generated to visualise the simultaneous effect of both technological factors on the viability of *Pseudomonas* spp., enabling the identification of critical regions for microbial growth or inhibition.

**Figure 2:**
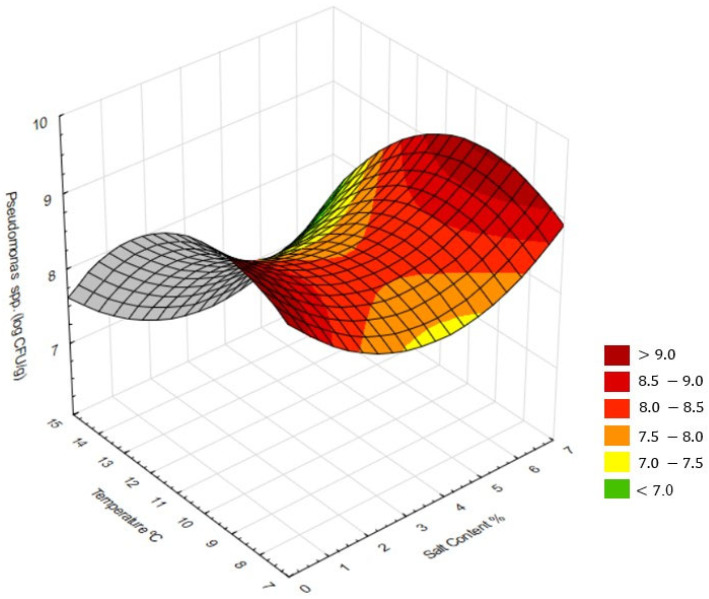
Response surface plots showing the combined effects of salt concentration and ripening temperature on microbial and physicochemical traits of cheeses after 20 days of ripening: counts of *Pseudomonas* spp. (log CFU/g).

RSM illustrated the combined effects of temperature and salt concentrations on microbial traits (Figure 2). *Pseudomonas* spp. viability was lowest at 14 °C with moderate salinity, displaying a “saddle-like” shape, indicating a non-linear interaction between temperature and salt concentration. At lower salt levels, increasing temperature promoted *Pseudomonas* spp. growth, whereas, at higher salt concentrations, the same temperature increase resulted in inhibition. Similar interactive effects were reported, showing that the combined influence of temperature and NaCl determines *Pseudomonas* spp. growth kinetics, with salt strongly increasing lag time and reducing growth rate [62]. This antagonistic interaction reflects the stimulatory effect of temperature on metabolism versus the osmotic stress imposed by salt.

### 3.2. Principal Component Analysis (PCA)

To comprehensively assess the influence of salt content and ripening temperature on the samples, PCA was applied to triplicate data collected after 20 days of ripening. Twelve attributes encompassing microbiological, physicochemical, and colour attributes were included: titratable acidity, fat, total solids (TS), total nitrogen (TN), water-soluble nitrogen (WSN), trichloroacetic acid-soluble nitrogen 12% (TCA-SN), hardness, and rind colour parameters (L*, a* and b*), as well as counts of *Pseudomonas* spp. and LAB. PCA allowed for dimensionality reduction and the identification of association patterns among variables, with only the first three principal components retained, since they exhibited eigenvalues greater than 1. These three components retained together 86.5% of the total variance (44.6% for the first, 33.2% for the second, and 8.6% for the third). Table 6 presents the eigenvalues, percentage of explained variance, and correlation coefficients between the original attributes and the first three principal components.

The first three principal components accounted for 86.5% of total variance (Table 6). PC1 strongly associated with proteolysis indices (WSN, TCA-SN, and maturation coefficient) and texture parameters. PC2 was mainly related to colour coordinates (L*, a*, and b*), fat content, and *Pseudomonas* spp. counts. While PC3 contributed marginally, explaining a smaller proportion of variance, it was negatively correlated with LAB counts.

**Figure 3:**
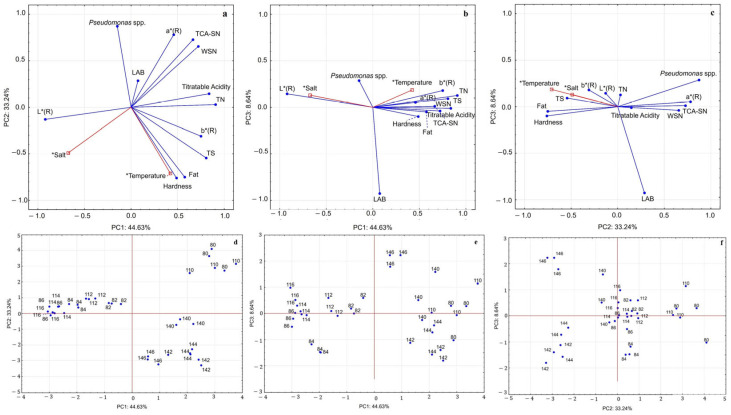
PCA output of cheese samples after 20 days of ripening. Variable projection: (**a**) PC1 vs. PC2, (**b**) PC1 vs. PC3, and (**c**) PC2 vs. PC3. Supplementary variables (*) are in red (salt concentration and ripening temperature). Samples projection: (**d**) PC1 vs. PC2, (**e**) PC1 vs. PC3, and (**f**) PC2 vs. PC3.

Score plots (Figure 3) showed a clear separation according to ripening temperature: cheeses matured at 14 °C clustered apart from those ripened at 8 °C and 11 °C, reflecting higher proteolysis and firmer texture. Salt concentration functioned as a secondary factor, differentiating low-salt (0% and 2%) from high-salt (4% and 6%) cheeses, although its effect was less pronounced than temperature.

## 4. Discussion

The application of response surface methodology (RSM) and principal component analysis (PCA) allowed the integration of microbial, physicochemical, textural, and chromatic data, offering a global perspective on how ripening temperature and salt concentration interact in shaping the quality of traditional ewe’s milk. The univariate analyses provided detailed insight into specific changes, while the multivariate approaches confirmed the overarching patterns. Together, these results demonstrate that temperature is the main determinant of microbial and biochemical dynamics, while NaCl exerts a non-linear modulatory role whose effect depend on microbial competition and curd matrix stability.

### 4.1. Microbiological Dynamics

From a microbiological perspective, *Pseudomonas* spp. counts remained high (>8 log CFU/g) across the salt and temperature conditions, confirming their resilience in dairy matrices. This persistence, even under hypersaline conditions, is consistent with previous reports indicating that *Pseudomonas fluorescens* tolerates 2.5–3.5% NaCl and survives during refrigerated storage [8,20]. The only condition where significant inhibition was observed was at 14 °C with 2% salt, where populations decreased to 6.58 log CFU/g. LAB consistently reached high levels (>8 log CFU/g) under all conditions, with maximum values observed at 8 °C and 4% salt. This dominance reflects their adaptive capacity and competitive role during ripening, both through acidification and the production of bacteriocins and antimicrobial metabolites [8,13,16]. Their tolerance to high salt concentrations further strengthens their competitive advantage within this ecosystem [63]. Although *Pseudomonas* spp. counts at 11 °C were comparable between cheeses produced with 0% and 2% salt, surface discolouration was only observed in the unsalted samples. This finding suggests that salt influenced bacterial physiology rather than overall population size. The absence of salt increases water activity and reduces osmotic stress, conditions known to enhance the metabolic activity and pigment synthesis of *Pseudomonas fluorescens*, has shown that water activity and osmotic stress modulate cellular respiration and secondary metabolite formation in dairy systems, supporting this interpretation [25,64,65].

Fungal counts ranged from moderate to high values (≈8 log CFU/g) depending on the combination of factors. The interaction between salt and temperature significantly influenced their growth, consistent with their role in lipolysis, the formation of aroma compounds, and the risk of technological defects [28].

### 4.2. Physicochemical and Proteolysis Composition

The titratable acidity showed significant variations (*p* < 0.05) among the different ripening conditions. Overall, samples with added salt displayed lower acidity values at the end of ripening, which is consistent with the expected behaviour: acidity tends to decrease during maturation due to lactic acid metabolism and the subsequent formation of neutral or alkaline compounds [60]. In salt-free cheeses, however, this decrease was less pronounced, resulting in higher final acidity values, reflecting greater microbial and fermentative activity. This pattern was also evident in the visual appearance of the cheese, as illustrated in Figure 1. Cheeses without added salt, particularly at 8 °C and 11 °C, showed more pronounced fermentative progression and acid release, whereas cheeses with higher NaCl contents exhibited more regular rinds and firmer pastes. This reflects the inhibitory effect of salt on microbial activity and acidification, in line with literature reports on enzymatic activity [29,32]. Fat content in samples ripened at 14 °C, regardless of salt level, was significantly higher (*p* < 0.05) than that of cheeses ripened at lower temperatures, and total solids followed the same trend. Cheeses ripened at 14 °C exhibited a drier and more homogeneous paste, while lower temperatures yielded reduced dry matter. The direct relationship between moisture loss and fat concentrations is well documented in the literature [29,32,35].

Total nitrogen (TN) showed significant differences (*p* < 0.05), with minimum values at 11 °C and 6% salt and maximum values at 14 °C and 0% salt. This pattern reflects the combined influence of temperature and salt on solid retention: at higher ripening temperatures, cheeses exhibited a more cohesive paste and higher protein expression (Figure 1), whereas excess salt promoted more compact and uniform cheeses, suggesting reduced degradation and greater inhibition of enzymatic and microbial activity [6,32]. The role of native microbiota in this dynamic has been widely emphasised as a determinant of proteolysis intensity [8,13]. TCA-SN/TN% values were significantly higher (*p* < 0.05) in cheeses ripened at 8 °C without salt, indicating that low ripening temperature combined with the absence of NaCl promoted proteolysis. These cheeses displayed a less consistent paste and more degraded appearance. Conversely, in high-salt combinations (≥6%), both WSN/TN% and TCA_SN/TN% values were significantly lower (*p* < 0.05), with firmer and more stable structures observed visually, confirming the inhibitory effect of salt and microbial influence on casein hydrolysis [8,16,29,60]. The WSN/TN%, an overall indicator of proteolysis extent, confirmed this trend. Higher values were observed under low-salinity conditions, particularly at lower ripening temperatures. In contrast, consistently lower values occurred under high-salt conditions, regardless of ripening temperature. Additionally, the lowest WSN/TN% values were recorded at 14 °C, evidencing both the role of salt and higher ripening temperature as inhibitors of proteolysis [33].

### 4.3. Texture Development and Colour Changes

Hardness varied significantly (*p* < 0.05), with values below 5 N recorded at ripening temperatures of 8 °C and 11 °C, reflecting a moister matrix (<total solids) and more advanced proteolysis (>WSN/TN%). In contrast, a marked increase was observed at 14 °C, confirming ripening temperature as the dominant factor [59,60]. Regarding the effect of salt, at each ripening temperature, the samples showing the highest hardness were those produced with 2% salt, suggesting optimal casein network compaction at moderate salt levels and a loss of firmness when salt was excessive [57,58]. Adhesiveness followed a similar pattern to hardness, with the most adhesive cheeses generally being those ripened at 14 °C and, within each temperature group, those produced with 2% and 4% salt. This reflects high cohesiveness and matrix retention, associated with the higher fat and total solids content already observed in Table 3. Visually, these cheeses showed a more compact and uniform appearance, in contrast with cheeses ripening at 8 °C and 11 °C, where more pronounced syneresis resulted in drier and less adhesive textures.

In terms of CIELab analysis, for the rind, cheeses ripened without salt at 8 °C and 11 °C exhibited lower luminosity (darker colouration), consistent with the higher acidity values already discussed. This darkening is associated with greater microbial prevalence, particularly the activity of *Pseudomonas* spp., which enhances pigment and oxidative compound formation. Conversely, under higher salinity conditions, cheeses appeared lighter and more homogeneous, reflecting the inhibitory effect of NaCl on microbial activity. The a* parameter confirmed this trend: reddish tones predominated under low-salt conditions with more intense fermentative activity, whereas, at higher ripening temperatures and with added salt, the colouration shifted toward more stable greenish tones. The b* parameter revealed an intensification of yellow hues in cheeses ripened at 14 °C, especially with moderate salinity, resulting in rinds that were visually lighter and cream yellowish. For the paste, luminosity also varied significantly but without a linear pattern, indicating that factors such as moisture or matrix density also influenced this parameter. The a* colour parameter was consistently negative, confirming greenish tones, less pronounced at 8 °C and 11 °C and more intense at 14 °C. The b* values indicated higher yellow intensity at elevated temperatures, while lower values predominated at 11 °C. Overall, the results show that ripening temperature exerted a stronger influence on colour tones (a* and b*) than salt concentration, while luminosity (L*) was less dependent on the experimental conditions.

### 4.4. Response Surface Analysis (RSM)

The RSM regarding the interaction between ripening temperature and salt concentration on the viability of *Pseudomonas* spp. (Figure 2) revealed a non-linear behaviour, with clear evidence of significant interaction between factors. Microbiological response, expressed as log CFU/g, showed higher values under two extreme conditions: higher temperatures combined with low salt levels and lower temperature associated with high salt concentrations. This topographical configuration suggests the presence of a stationary point, where bacterial reduction occurs only within an intermediate range of conditions, approximately between 10 and 12 °C and 3 and 4% salt. The interaction analysis indicates that optimal inhibitory effect does not result from the maximisation of a single factor but rather from their balanced combination. Moderate salt levels coupled with controlled ripening temperature reduced the growth rate without severely compromising the technological and sensory properties of the cheese. From a biological perspective, this “saddle-like” surface reflects the antagonistic interaction between temperature and salinity on *Pseudomonas* spp. physiology. Increasing temperature enhances microbial metabolism and enzymatic activity, whereas higher salt concentrations impose osmotic stress and decrease water activity, limiting bacterial proliferation. Similar interactions between salt and temperature have been observed in other *Pseudomonas* species, where salinity markedly extended lag time and reduced growth at low temperatures. In dairy systems, moderate salt levels were shown to limit *Pseudomonas* spp. development while preserving cheese quality, supporting the idea that around 2% salt and 14 °C provide balanced conditions for proper maturation without microbial defects.

However, it is noteworthy that, even under the most favourable conditions within this surface, counts remained relatively high (>7 log CFU/g). This pattern is consistent with previous reports, where *Pseudomonas fluorescens* demonstrated both high tolerance to osmotic stress and the ability to grow under refrigerated environments. Strains of this genus can remain viable at NaCl concentrations above 2.5–3.5% through osmotic homeostasis mechanisms that confer resilience in salted matrices [17]. Its psychotropic nature further enables growth at low temperature, even though at reduced rates, while, at higher temperatures (12–14 °C), cell multiplication intensifies together with the synthesis of thermostable extracellular enzymes, such as the protease AprX and various lipases, which are responsible for sensory and physicochemical alterations in cheese. These results reinforce that the growth of *Pseudomonas* spp. in cheese depends on the interactions of multiple environmental factors, including temperature, salinity, water activity, and microbial competition [24,26].

### 4.5. Principal Component Analysis (PCA)

The projection on the PC1 vs. PC2 plane (Figure 3a,d) highlighted three main clusters: (i) samples at 8 °C and 11 °C without salt, which showed strong proteolytic activity (high WSN and TCA-SN), colour defects associated with an increase in the a* (R) parameter, and higher incidence of *Pseudomonas* spp. These conditions favoured excessive matrix degradation, compromising quality. (ii) Samples ripened at 14 °C (0–6% salt), which were also proteolyzed but displayed different features: greater hardness and higher total solids, more pronounced characteristic yellow colouration (higher b* values), and less evidence of colour defects (lower a* values). These conditions suggested that the higher temperature accelerated ripening but the effect of salt partially moderated microbial activity. (iii) Samples at 8 °C and 11 °C with salt levels between 2% and 6%, which were mainly characterised by lighter rinds (higher L*), lower proteolysis intensity, and reduced developments of undesirable microorganisms (*Pseudomonas* spp.) This group highlighted the productive role of salt, which reduced microbial activity and stabilised visual and structural characteristic compared with group (i). The inclusion of PC3 (Figure 3b,c,e,f) reinforced these conclusions, showing that the determining variable on this axis was the presence of LAB, which exhibited an opposite trend to *Pseudomonas* spp. LAB proved highly resilient, adapting well to different salt levels. Only at lower temperature was a slight inhibitory effect observed, possibly due to competition with *Pseudomonas* spp. This pattern confirms the antagonistic relationship between *Pseudomonas* spp. and LAB during ripening, a phenomenon widely reported in raw milk cheeses, where LAB act as a natural barrier against contaminants. However, at low temperatures, their action may be limited by the competitiveness of *Pseudomonas* spp., which can grow and cause rind alterations [8,13,16,17,58].

## 5. Conclusions

Overall, the results confirm that the quality and stability of ripened ewe’s milk cheese arise from a dynamic balance between technological factors (salt and temperature) and ecological interactions (microbial competition). From a microbiological perspective, *Pseudomonas fluorescens* showed high resilience, maintaining viability even under high salinity, demonstrating that NaCl alone does not represent an effective barrier. On the other hand, the autochthonous microbiota played a decisive role: LAB, through matrix acidification and the production of antimicrobial metabolites, partially limited the expansion of *Pseudomonas* spp., while fungi, emerging later, diversified metabolism and contributed to the sensory profile but also competed with LAB for substrates. This dynamic reinforced the idea that ecological competition may represent a more relevant barrier than salt addition alone. From a chemical and structural perspective, ripening temperature emerged as the most critical factor in controlling *Pseudomonas* spp. At 14 °C, proteolysis was more intense; however, higher total solids content resulted in a firmer and more compact texture. Regarding colour, the combined effect of low temperatures and salt addition accentuated rind discolouration defects caused by *Pseudomonas* spp. In terms of scientific and technological contribution, this study emphasises that, while NaCl is essential to modulate acidity and contribute to texture, it is insufficient to control undesirable populations such as *Pseudomonas fluorescens*. Instead, the combined management of salinity, temperature, and microbial ecology emerges as a key strategy to ensure microbiological stability and sensory quality in traditional raw ewe’s milk cheeses. Based on this study, these findings can be synthesised into four main conclusions:

1. Low temperatures without salt enhance colour defects caused by the growth of undesirable microorganisms, particularly *Pseudomonas* spp.
2. Very high salt levels, although contributing to partial control of *Pseudomonas* spp., delay ripening and result in cheeses with a pale, uncharacteristic appearance.
3. Higher ripening temperatures accelerate both proteolysis and dehydration, leading to harder cheeses.
4. Moderate salt levels (2%) combined with higher ripening temperatures promote more controlled maturation, with a uniform characteristic appearance and no structural defects.

## Figures and Tables

**Table 1:** Physicochemical and microbiological characterisation means and standard deviation (within brackets) of raw ewe’s milk used for cheese production.

	Raw Milk
NaCl Content % (m/m)	0.23 (0.01)
pH	6.62 (0.03)
Titratable Acidity (mL NaOH 0.1 N/L)	18.00 (0.00)
Protein % (m/m)	2.52 (0.02)
Fat % (m/m)	6.21 (0.04)
Lactose % (m/m)	4.95 (0.01)
Cryoscopic Point (°C)	−0.516 (0.000)
Total Solids % (m/m)	13.77 (0.04)
Non-fat Solids % (m/m)	9.05 (0.01)
LAB (log CFU/mL)	4.38 (0.09)
*Pseudomonas* spp. (log CFU/mL)	7.13 (0.02)

**Table 2:** Means and standard deviation (within brackets) of the log counts of different microbial groups analysed in the cheese samples after 20 days of ripening under different salt concentrations and temperatures. Results are expressed as mean log CFU/g.

Ripening Temperature	Salt Concentration	*Pseudomonas* spp.	LAB	Yeasts and Molds
8 °C	0%	9.30 (0.44) ^ab^	8.81 (0.23) ^ab^	8.56 (0.59) ^ab^
	2%	8.75 (0.25) ^abc^	8.60 (0.08) ^abc^	9.05 (0.20) ^a^
	4%	8.70 (0.20) ^abc^	8.98 (0.04) ^a^	8.69 (0.06) ^ab^
	6%	8.57 (0.17) ^abc^	8.65 (0.08) ^abc^	8.67 (0.16) ^ab^
11 °C	0%	9.29 (0.25) ^ab^	8.62 (0.21) ^abc^	5.55 (0.18) ^c^
	2%	8.92 (0.09) ^ab^	8.60 (0.11) ^abc^	8.29 (0.57) ^ab^
	4%	8.51 (0.20) ^abc^	8.60 (0.03) ^abc^	8.19 (0.06) ^ab^
	6%	8.48 (0.11) ^bc^	8.41 (0.11) ^bc^	8.21 (0.13) ^ab^
14 °C	0%	8.77 (0.15) ^abc^	8.51 (0.22) ^abc^	8.10 (0.27) ^ab^
	2%	6.58 (0.15) ^e^	8.74 (0.09) ^ab^	7.69 (0.25) ^b^
	4%	7.33 (0.30) ^de^	8.82 (0.12) ^ab^	8.78 (0.15) ^ab^
	6%	7.95 (0.20) ^cd^	8.09 (0.08) ^c^	8.61 (0.15) ^ab^

Means in the same column marked with different letters are significantly different (*p* < 0.05, *n* = 3, Scheffé test). LAB: lactic acid bacteria.

**Table 3:** Means and standard deviation (within brackets) of the physicochemical parameters (pH, titratable acidity, fat, and total solids) of cheese samples after 20 days of ripening under different salt concentrations and temperatures.

Ripening Temperature	Salt Concentration	pH	Titratable Acidity (mL NaOH 0.1 N/100 g)	Fat % (m/m)	Total Solids % (m/m)
8 °C	0%	5.43 (0.05) ^ab^	15.73 (0.46) ^a^	28.17 (1.26) ^e^	60.22 (1.45) ^b^
	2%	5.33 (0.01) ^b^	7.20 (0.80) ^e^	27.83 (0.29) ^e^	54.33 (0.90) ^c^
	4%	5.20 (0.02) ^b^	9.07 (0.46) ^cde^	29.00 (1.00) ^de^	50.61 (1.25) ^cde^
	6%	5.04 (0.05) ^b^	8.27 (0.46) ^de^	26.17 (0.76) ^e^	47.34 (0.95) ^e^
11 °C	0%	5.43 (0.04) ^ab^	16.27 (2.44) ^a^	27.83 (1.04) ^e^	59.56 (1.20) ^b^
	2%	5.32 (0.05) ^b^	8.27 (0.46) ^de^	26.50 (1.00) ^e^	53.37 (1.83) ^cd^
	4%	4.91 (0.02) ^b^	8.80 (0.80) ^de^	26.33 (0.58) ^e^	49.02 (0.76) ^de^
	6%	5.13 (0.06) ^b^	10.40 (0.00) ^b–e^	25.50 (0.00) ^e^	49.01 (0.04) ^de^
14 °C	0%	6.25 (0.70) ^a^	11.20 (0.80) ^bcd^	32.00 (0.50) ^cd^	67.99 (1.63) ^a^
	2%	5.05 (0.04) ^b^	13.60 (1.39) ^ab^	35.17 (1.61) ^bc^	69.49 (2.35) ^a^
	4%	4.95 (0.04) ^b^	12.80 (0.00) ^abc^	39.33 (0.76) ^a^	71.53 (0.30) ^a^
	6%	5.03 (0.03) ^b^	11.73 (0.46) ^bcd^	37.00 (0.87) ^ab^	71.29 (0.78) ^a^

Means in the same column marked with different letters are significantly different (*p* < 0.05, *n* = 3, Scheffé test).

**Table 4:** Means and standard deviation (within brackets) of the physicochemical parameters (total protein content and nitrogen fractions (water-soluble nitrogen and trichloroacetic-soluble nitrogen)) of cheese samples after 20 days of ripening under different salt concentrations and temperatures.

Ripening Temperature	Salt Concentration	TN % (m/m)	WSN/TN %	TCA-SN/TN %
8 °C	0%	4.64 (0.09) ^ab^	53.99 (0.46) ^a^	21.85 (2.03) ^a^
	2%	3.84 (0.06) ^d^	39.17 (1.78) ^b^	10.93 (1.92) ^b^
	4%	3.02 (0.14) ^e^	35.76 (1.57) ^bcd^	8.70 (0.37) ^bc^
	6%	2.74 (0.06) ^e^	28.83 (2.57) ^cde^	3.05 (0.23) ^d^
11 °C	0%	4.55 (0.14) ^abc^	51.27 (4.23) ^a^	21.73 (2.59) ^a^
	2%	3.87 (0.09) ^d^	34.17 (0.66) ^bcd^	8.69 (0.87) ^bc^
	4%	2.76 (0.09) ^e^	37.75 (1.45) ^bc^	6.55 (1.14) ^bcd^
	6%	2.70 (0.11) ^e^	27.97 (2.59) ^de^	3.48 (0.72) ^d^
14 °C	0%	5.13 (0.15) ^a^	32.17 (2.10) ^bcd^	9.95 (0.79) ^b^
	2%	4.25 (0.30) ^bcd^	31.83 (2.49) ^bcd^	6.35 (0.24) ^bcd^
	4%	4.15 (0.26) ^bcd^	27.58 (3.75) ^de^	4.68 (0.81) ^cd^
	6%	4.02 (0.02) ^cd^	20.22 (1.39) ^e^	2.24 (0.26) ^d^

Means in the same column marked with different letters are significantly different (*p* < 0.05, *n* = 3, Scheffé test). TN: total nitrogen; WSN/TN: water-soluble nitrogen relative to TN; TCA-SN/TN: trichloroacetic acid-soluble nitrogen relative to TN.

**Table 5:** Means and standard deviation (within brackets) of the colour and texture properties analysed of the cheese samples after 20 days of ripening under different salt concentrations and temperatures.

Ripening °C	Salt %	Mechanical Properties		CIELab System					
				Rind			Paste		
		Hardness (N)	Adhesiveness (−Ns)	L*	a*	b*	L*	a*	b*
8 °C	0%	3.05 ^c^ (0.29)	12.99 ^c^ (1.01)	67.97 ^d^ (2.69)	5.21 ^a^ (1.21)	16.55 ^ab^ (1.74)	98.82 ^a^ (2.84)	−2.37 ^bcd^ (0.21)	13.40 ^abc^ (0.62)
	2%	4.43 ^c^ (0.10)	14.26 ^c^ (3.54)	75.54 ^bc^ (1.26)	−0.69 ^c^ (0.11)	15.94 ^ab^ (2.09)	80.29 ^bcd^ (0.99)	−2.29 ^a–d^ (0.06)	13.53 ^abc^ (0.57)
	4%	1.85 ^c^ (0.13)	9.14 ^c^ (0.74)	83.49 ^a^ (0.90)	−1.34 ^c^ (0.34)	12.33 ^b^ (1.40)	87.43 ^bc^ (0.69)	−1.85 ^a–d^ (0.09)	11.83 ^a–e^ (0.17)
	6%	3.19 ^c^ (0.15)	11.80 ^c^ (1.12)	84.85 ^a^ (0.63)	−1.28 ^c^ (0.34)	12.66 ^b^ (0.40)	88.35 ^ab^ (0.62)	−1.31 ^a^ (0.16)	8.93 ^de^ (0.30)
11 °C	0%	3.13 ^c^ (0.73)	10.68 ^c^ (1.64)	60.39 ^e^ (2.40)	2.58 ^b^ (1.64)	16.61 ^ab^ (2.51)	71.62 ^d^ (3.76)	−1.70 ^a–d^ (0.10)	7.79 ^e^ (2.12)
	2%	2.95 ^c^ (0.31)	16.44 ^c^ (2.09)	80.76 ^ab^ (0.64)	−0.52 ^c^ (0.20)	11.45 ^b^ (0.53)	81.55 ^bcd^ (0.48)	−1.99 ^a–d^ (0.37)	12.31 ^a–d^ (1.04)
	4%	2.23 ^c^ (0.19)	10.13 ^c^ (1.77)	85.75 ^a^ (1.49)	−1.15 ^c^ (0.35)	12.82 ^b^ (0.48)	86.98 ^bc^ (1.85)	−1.53 ^abc^ (0.05)	10.30 ^b–e^ (0.48)
	6%	3.17 ^c^ (0.45)	11.04 ^c^ (0.54)	85.83 ^a^ (0.44)	−1.21 ^c^ (0.08)	11.12 ^b^ (1.13)	86.62 ^bc^ (5.40)	−1.45 ^ab^ (0.22)	9.67 ^cde^ (1.00)
14 °C	0%	26.33 ^b^ (6.60)	38.62 ^c^ (7.70)	69.74 ^cd^ (1.29)	−1.83 ^c^ (0.11)	16.30 ^ab^ (1.61)	72.83 ^d^ (1.58)	−2.06 ^a–d^ (0.37)	14.56 ^ab^ (1.31)
	2%	48.71 ^a^ (2.10)	123.96 ^ab^ (8.52)	66.48 ^de^ (3.79)	−2.24 ^c^ (0.43)	15.13 ^ab^ (2.03)	72.90 ^d^ (4.73)	−2.47 ^cd^ (0.38)	13.80 ^abc^ (1.55)
	4%	20.39 ^b^ (2.92)	97.79 ^b^ (15.54)	69.43 ^cd^ (0.50)	−1.03 ^c^ (0.43)	20.58 ^a^ (0.51)	76.94 ^cd^ (2.05)	−2.56 ^d^ (0.26)	15.46 ^a^ (0.20)
	6%	26.76 ^b^ (4.32)	158.60 ^a^ (25.87)	81.25 ^ab^ (1.11)	−1.58 ^c^ (0.33)	18.81 ^a^ (1.03)	84.28 ^bc^ (1.31)	−1.51 ^abc^ (0.28)	12.67 ^a–d^ (1.33)

Means in the same column marked with different letters are significantly different (*p* < 0.05, *n* = 3, Scheffé test).

**Table 6:** PCA summary table of ripened sheep’s milk cheeses after 20 days of ripening under different salt concentrations and temperatures. The first three components (eigenvalues > 1) explained 86.5% of the variance. The plot displays the projection of microbiological, physicochemical, and colour attributes, evidencing their contribution to the distribution of samples in the multivariate space.

Component	PC1	PC2	PC3
Titratable Acidity	0.84 *	0.15	−0.01
Fat	0.58	−0.75 *	−0.05
TS	0.81 *	−0.54	0.09
TN	0.91 *	0.03	0.13
WSN	0.72 *	0.66	−0.04
TCA-SN	0.66	0.73 *	0.01
Hardness	0.49	−0.76*	−0.1
L* (R)	−0.92 *	−0.13	0.15
a* (R)	0.46	0.78 *	0.05
b* (R)	0.75 *	−0.31	0.18
*Pseudomonas* spp. count	−0.15	0.87 *	0.29
LAB count	0.07	0.29	−0.93 *
Eigenvalue	5.36	3.99	1.04
% Variance	44.63	33.24	8.64
% Cumulative variance	44.63	44.63	86.51

Significant correlations are marked with a *, r > 0.7.

## Data Availability

Data will be made available on request.
